# Appearance of retinal arterial macroaneurysms in patients using swept-source optical coherence tomographic angiography

**DOI:** 10.1186/s12886-023-03016-x

**Published:** 2023-06-16

**Authors:** Yi Song, Weican Zhang, Suqin Yu, Yuanyuan Gong

**Affiliations:** 1 Department of Ophthalmology, Shanghai Municipal Hospital of Traditional Chinese Medicine, Shanghai University of Traditional Chinese Medicine, Shanghai, China; 2 Department of Ophthalmology, Shanghai First People’s Hospital), Shanghai General Hospital, Shanghai Jiao Tong University School of Medicine, Shanghai, China; 3 National Clinical Research Center for Eye Diseases, Shanghai, China; 4 Shanghai Key Laboratory of Ocular Fundus Diseases, Shanghai, China; 5 Shanghai Engineering Center for Visual Science and Photomedicine, Shanghai, China; 6 Shanghai Engineering Center for Precise Diagnosis and Treatment of Eye Diseases, Shanghai, China; 7 Shanghai Clinical Research Center for Eye Diseases, Shanghai, China; 8 Shanghai Key Clinical Specialty, Shanghai, China

**Keywords:** Retinal arterial macroaneurysms, Optical coherence tomography angiography, Fluorescein angiography

## Abstract

**Background:**

Retinal arterial macroaneurysm (RAM) is a common clinical disease leading to vision loss in elderly individuals. The appropriate interpretation of swept-source optical coherence tomographic angiography (SS-OCTA), a noninvasive examination, is easy and convenient for detecting the status of RAMs and guiding treatment.

**Methods:**

The objectives of this study were to describe the morphologic characteristics of RAMs using SS-OCTA and to observe whether there are differences in the morphologies of RAMs between SS-OCTA and fundus fluorescein angiography (FFA), before and after treatment. We retrospectively evaluated twenty-two eyes of 22 patients who were diagnosed with RAMs. All patients underwent a complete ophthalmologic examination, including a review of medical records, best-corrected visual acuity (BCVA), fundus photography, FFA and SS-OCTA. RAMs were recorded by SS-OCTA before any treatment or observation decisions were made. The morphologic findings of the RAMs on SS-OCTA were investigated.

**Results:**

On SS-OCTA, RAMs can show local dilatation or an irregular linear blood flow signal, and the dilated cystic lumen may show thrombosis with a low reflection signal. After treatment, the shape of the RAMs will show reactive changes. The findings on SS-OCTA are not very consistent with those on FFA.

**Conclusions:**

The same RAM may have different manifestations on OCTA and FFA, and OCTA can more conveniently reflect the changes in blood flow signals and treatment response of RAMs.

## Background

*Retinal arterial macroaneurysm* (RAM) was first reviewed by Robertson in 1973 [1], and the prevalence of the RAM is reported as 1 in 1,500 to 4,500 people [2, 3]. RAM is described as an acquired, focal dilation of a retinal artery, typically occurring within the first three bifurcations of the central retinal artery. Although RAMs are usually a solitary, unilateral finding, multiple RAMs may be observed in 15–20% of cases and bilateral disease occurs in up to 10% of cases [4–7]. In agreement with most studies reporting a predilection age range of 66 to 74, RAMs are most commonly observed in elderly females, with a female preponderance of approximately 70%. Among the common systemic conditions associated with RAMs, hypertension ranks first and is present in approximately 75% of presenting patients [8, 9], followed by arteriosclerosis and abnormal lipid levels. In recent years, the pathophysiology behind the formation of RAMs has been partly understood. Inflammation, alterations of pericytes, or dysregulated extracellular matrix turnover which are associated with angiotensin II, may result in weakness of the arterial wall where RAMs protrude out [10]. As the resolution of optical coherence tomography (OCT) has improved, spectral-domain optical coherence tomography (SD-OCT) is more widely used to observe RAMs in different states, and these scans often reveal RAMs with hyperintense walls encompassing a hypointense lumen.

Clinically there is a lack of effective treatment methods, although with the level of diagnosis and treatment, the clinical manifestations of the disease are diverse, and the personalized treatment options are not the same. Swept-source optical coherence tomography angiography (SS-OCTA) is a common inspection method for fundus diseases. SS-OCTA is noninvasive and has high resolution. It can identify the movement information of retinal and choroidal blood flow and help clinicians understand the situation of fundus diseases [11, 12]. RAMs can be identified and diagnosed by angiography and the fundus morphology. However, there are few reports on the images on SS-OCTA, and there is a lack of research on the comparison with FFA morphology. The purpose of this study was to observe the different manifestations of SS-OCTA images of a series of patients under different intervention conditions, and to determine the differences between SS-OCTA and FFA. On SS-OCTA, the blood flow properties of RAMs in patients have been described. Although we traditionally believe that polyps are a saccular structure, they vary on SS-OCTA. The morphology of RAMs is described as a local bulge on the artery, which is also considered to be an enlarged structure on pathology. A cystic structure is displayed on FFA, but there are few studies on whether the structures displayed on SS-OCTA is completely consistent with those on FFA and whether SS-OCTA can exhibit the evolution of RAMs to guide the diagnosis and treatment, which are the observation purposes of this study.

## Methods

We retrospectively evaluated patients diagnosed with RAMs at the Department of Ophthalmology at Shanghai General Hospital, Shanghai, China from May 1, 2020, to July 30, 2021. The Ethics Committee of the Shanghai General Hospital approved this retrospective study, and determined that informed consent was not required, as all data were collected and analyzed in de-identified fashion. All investigations followed the tenets of the Declaration of Helsinki.

The inclusion criteria for the study were RAMs confirmed by at least 2 experienced ophthalmologists (S.Y. and Y.G.). ​The diagnosis of RAMs is based on the clinical presentation of the patients, and the dominant features, which are – abnormal vessel structure, hemorrhage or exudate, and its location on the retina, and these are expected to alter the differential diagnosis considered in each case. The exclusion criteria were severe media opacity, previous vitrectomy, presence of hemorrhage that prevented adequate FFA or SS-OCTA examinations, diabetic retinopathy, and the presence of other concomitant retinal diseases.

All patients underwent a complete ophthalmologic examination, including a review of medical records, best-corrected visual acuity, fundus photography (Visucam 200 digital fundus camera; Carl Zeiss Meditec AG), and simultaneous FFA (Spectralis; Heidelberg Engineering, Inc.), and SS-OCTA (VG200D; SVision Imaging, Ltd., Luoyang, China). SS-OCTA was performed in all patients before any treatment or observation decisions were made. SS-OCTA was performed using 3 × 3-mm and 6 × 6-mm macular raster scans centered on the lesion in all cases. The segmentation boundaries were then manually adjusted to optimally visualize the RAMs. The data collected from each patient included their history of eye diseases, treatments, and interpretations of their fundus photographic, and their FFA and SS-OCTA images. The SS-OCTA images were overlaid on the magnified FFA images to determine the position of the RAMs.

## Results

The 22 Asian patients included 18 women (81.82%) and 4 men (18.18%). The mean age was 71.6 (9.6) years, and the mean visual acuity before treatment for all patients was 0.913 ± 0.857 logMAR. Twenty-two eyes underwent imaging and were diagnosed with RAM. The demographic and clinical characteristics of the patients are summarized in the Table 1. A total of 7 eyes were treatment-naive, 6 received photocoagulations, 3 received single or multiple injections of vascular endothelial growth factor (VEGF) inhibitors, and 6 underwent a combination of anti-VEGF treatment and photocoagulation.

**Table 1:** Patient Information Summary

Patient No./Sex/Age, y	Hypertension	Eye	Course of Disease	Treatment
1/F/76s	Yes	Left	1 m	None
2/F/58s	Yes	Right	2 m	1 Photocoagulation
3/F/72s	No	Right	4 m	4 Anti-VEGF injections and 2 Photocoagulations
4/F/57s	Yes	Left	2w	2 Anti-VEGF injections and 1 Photocoagulation
5/F/77s	Yes	Left	3 m	None
6/F/80s	No	Left	1 m	1 Photocoagulation
7/F/80 s	Yes	Left	2 m	None
8/M/69s	Yes	Left	2w	None
9/F/81s	No	Right	19 m	3 Anti-VEGF injections and 5 Photocoagulations
10/M/74s	Yes	Left	4 m	4 Anti-VEGF injections, 1Photocoagulation and 1 Micropulse laser
11/F/85s	Yes	Right	2 m	2 Anti-VEGF injections and 1 Photocoagulation
12/F/72s	Yes	Left	2w	1 Photocoagulation
13/F/55s	No	Left	2w	1 Photocoagulation
14/F/85s	No	Left	6 m	1 Photocoagulation
15/M/58s	Yes	Right	2 m	2 Anti-VEGF injections and 1 Photocoagulation
16/F/70s	Yes	Right	1 m	1 Photocoagulation
17/M/58s	Yes	Left	1 m	None
18/F/76s	Yes	Right	1d	None
19/F/ 82s	No	Right	2w	None
20/F/ 63s	Yes	Right	1w	1 Anti-VEGF injection
21/F/79 s	Yes	Right	2w	1 Anti-VEGF injection
22/F/ 69s	Yes	Right	1w	5 Anti-VEGF injections

Abbreviations: VEGF, vascular endothelial growth factor

Note: Course of Disease means the time from the onset of symptoms until the first visit

### Patient 14

**Fig. 1:**
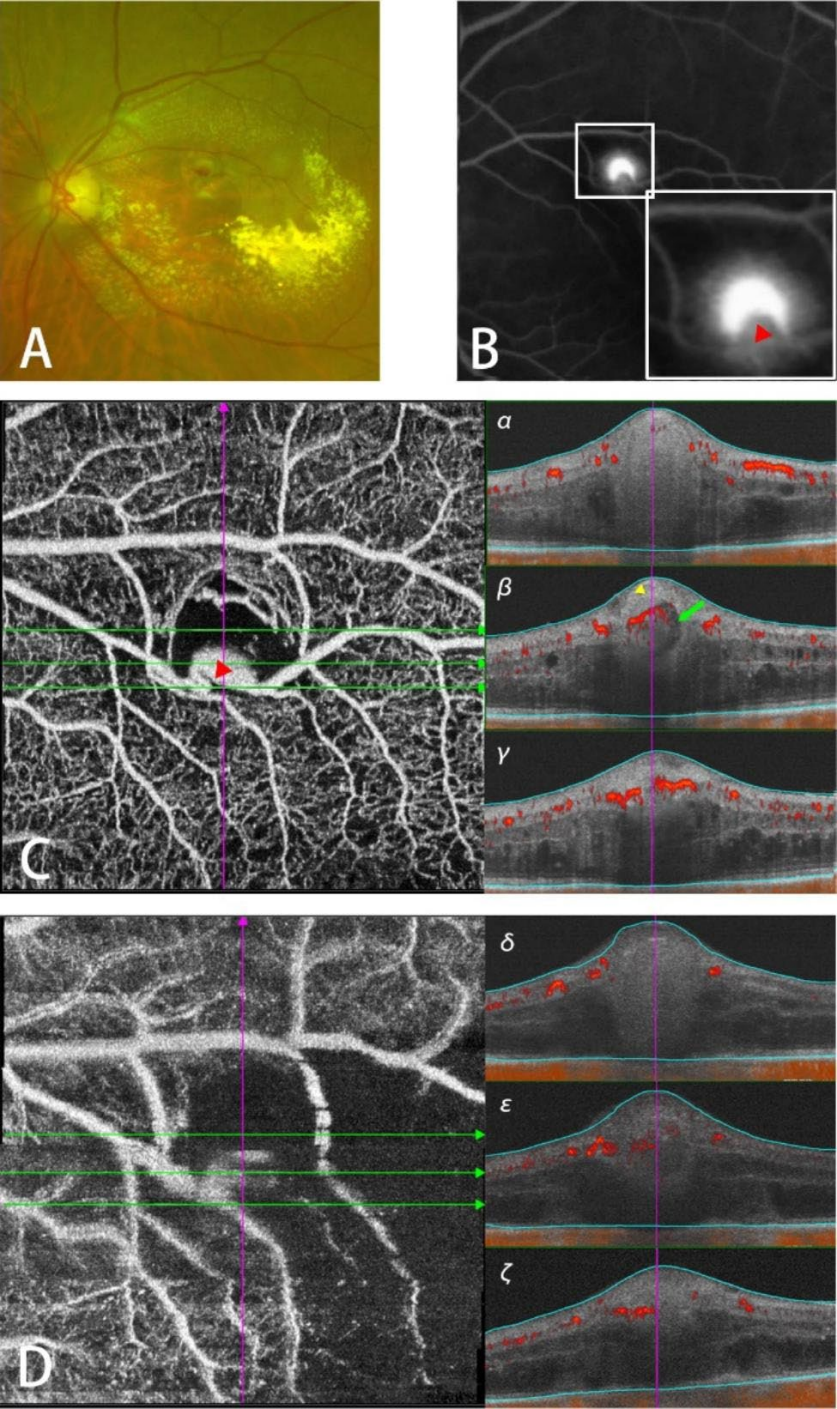
Multimodal Imaging in Patient 14 **A**: Fundus photography before treatment. **B**: FFA before treatment. The image in the lower white box is the enlarged image of RAM. Red triangle: the same lesion on C. **C**: SS-OCTA before treatment, **α-γ**: B-scan images captured at the green lines on C respectively from top to bottom. **Yellow triangle**: vascular wall. **Green arrow**: lumen. **D**: SS-OCTA taken immediately after photocoagulation. **δ-ζ**: B-scan images captured at the green lines on D respectively from top to bottom

An 85-year-old female diagnosed with RAM received 1 photocoagulation, and her BCVA before treatment was 20/200 (Fig. 1). Fundus photography showed a RAM above the macula (Fig. 1A). FFA revealed a saccular lesion with late-phase fluorescein leakage (Fig. 1B). The SS-OCTA taken before treatment revealed a locally enlarged, twisted blood vessel that was markedly smaller than the spherical bulge on FFA (Fig. 1C). The B-scan images α, β, γ were respectively captured from the green lines from top to bottom on image C. So were the images δ, ε, ζ on image D. The top line crosses the part of macroaneurysm which was not visualized on SS-OCTA, and the corresponding B-scan α revealed a dense and uniform reflection with a barely seen blood signal indicating the formation of blood turbulence or thrombus in that area. By scanning through the hyperintense lesion on the SS-OCTA, the image β from the middle line showed the comparatively hyper-reflective vascular wall .Inside it, the blood took on an uneven reflection with a masking effect on the B-scan. Immediately after photocoagulation, the SS-OCTA showed expansion of the nonperfusion area, including both the part of the RAM and downstream capillary (Fig. 1D). Compared with image β, the blood signal was decreased in the same place (Fig. 1Dε). Her BCVA one month after treatment was 20/200.

### Patient 13

**Fig. 2:**
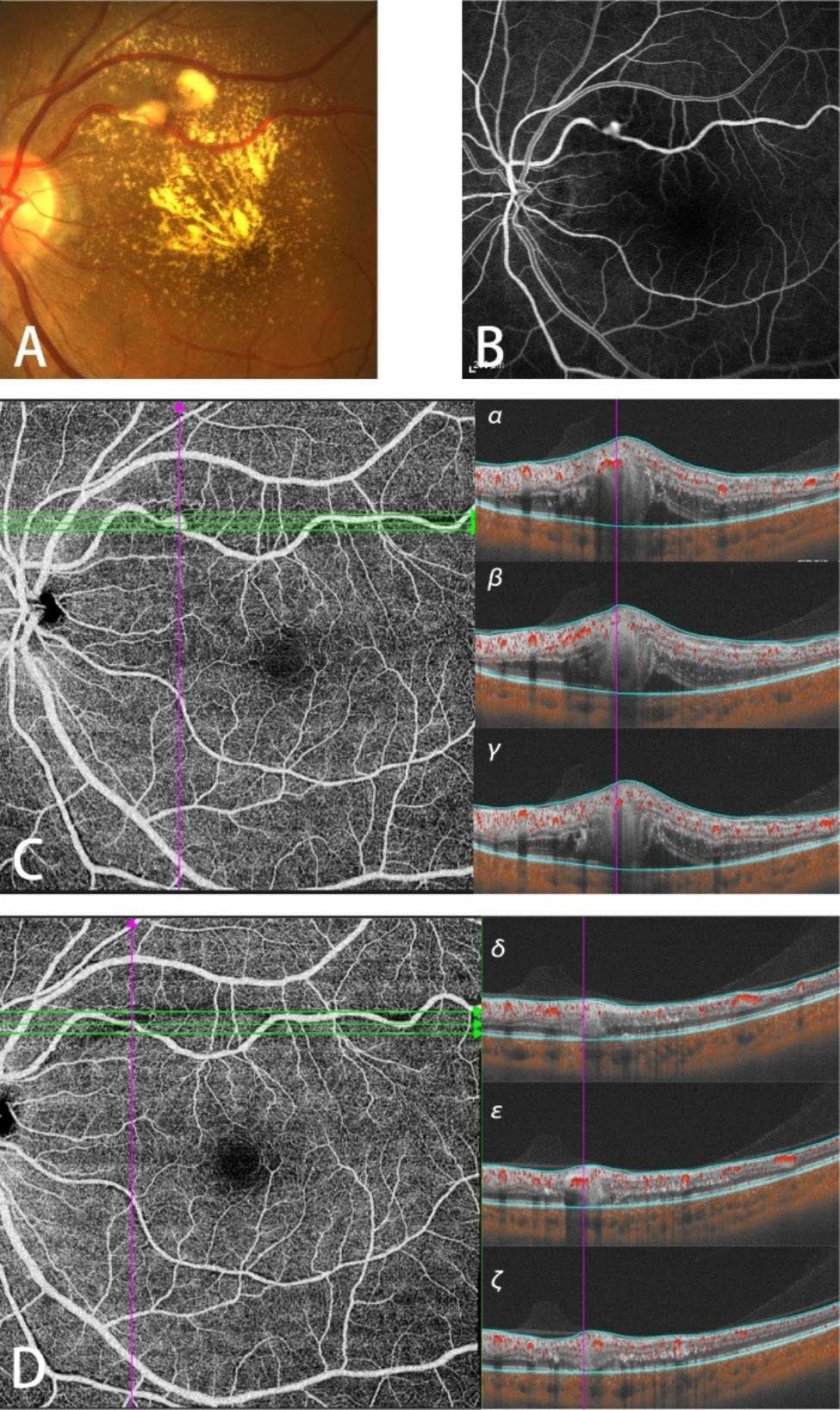
Multimodal Imaging in Patient 13 **A**: Fundus photography before treatment. **B**: FFA before treatment. **C**: SS-OCTA before treatment, **α-γ**: B-scan images captured at the green lines on C respectively from top to bottom. **D**: SS-OCTA taken 22 days after photocoagulation. **δ-ζ**: B-scan images captured at the green lines on D respectively from top to bottom

A 55-year-old woman received 1 photocoagulation and was followed up 22 days later (Fig. 2), whose BCVA before treatment was 20/400. Fundus photography of the left eye was taken before the treatment (Fig. 2A). The lesion had the typical fluorescent-stained features of RAMs (Fig. 2B). With the appearance of the stained vessel which RAM ever protruded from on the twenty-second day after treatment on OCTA, the photocoagulation successfully enclosed the macroaneurysm, and peripheral tissue ischemia was avoided (Fig. 2D). Similarly, B-scan images demonstrate the flow of RBCs rather than the structure of macroaneurysms (Fig. 2Cσβγ, Fig. 2Dδεζ).Her BCVA was ended up with 20/100 22 days after treatment.

### Patient 10

**Fig. 3:**
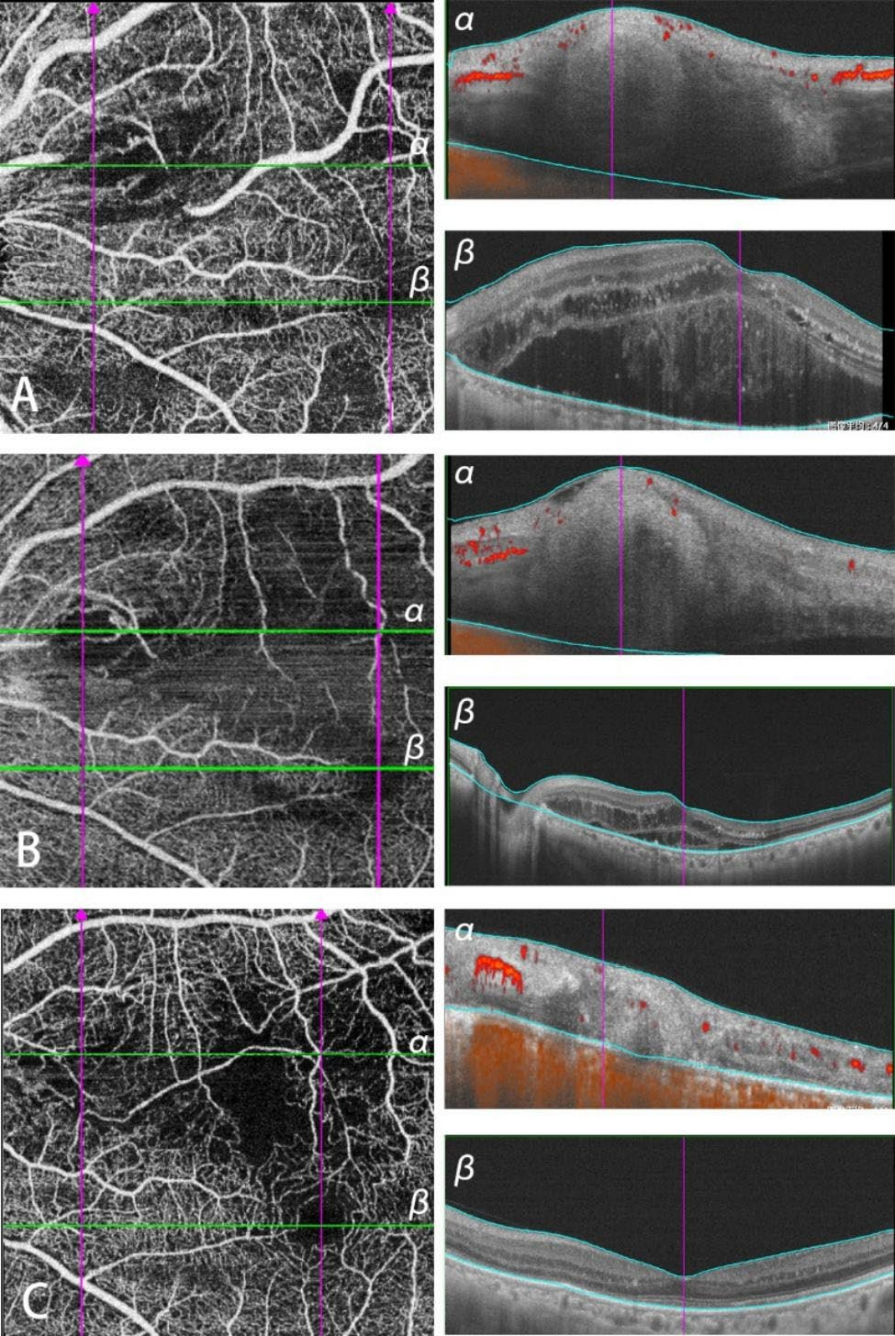
Multimodal Imaging in Patient 10 **A**: SS-OCTA taken 29 days after 4 anti-VEGF injections. **α**: B-scan image captured at the green line α on A. **β**: B-scan image captured at macula. B: SS-OCTA taken 36 days after photocoagulation. **α**: B-scan image captured at the green line α on A. **β**: B-scan image captured at macula. **C**: SS-OCTA taken 7months after micropulse laser. **α**: B-scan image captured at the green line α on A. **β**: B-scan image captured at macula

After the onset of symptoms, a 74-year-old male received a combinational treatment of anti-VEGF injections once a month for 4 months, then photocoagulation and micropulse laser were given respectively over the next two months. The RAM was located in the patient’s left eye, supranasal to the macular area. The image taken 29 days after the 4 anti-VEGF injections showed the absence of a blood flow signal at the RAM, while the capillaries compensated for the lack of circulation, making the blood flow signal to be detected in the downstream vessel (Fig. 3A). B-scan images were captured at the green line on A (Fig. 3Aα) and macula (Fig. 3Aβ) respectively showing exudation of the lesion affected the macula. The interruption of the blood flow signal and the nonperfusion area downstream was examined by SS-OCTA 36 days after photocoagulation (Fig. 3B) and the macular edema was decreased (Fig. 3Bβ). Seven months after micropulse laser, the patient was followed up and had another SS-OCTA, which revealed the formation of collateral circulation relieving the lack of local blood supply (Fig. 3C). Before and three months after the last treatment, the BCVA of the patient stabilized at 20/200.

### Patient 9

**Fig. 4:**
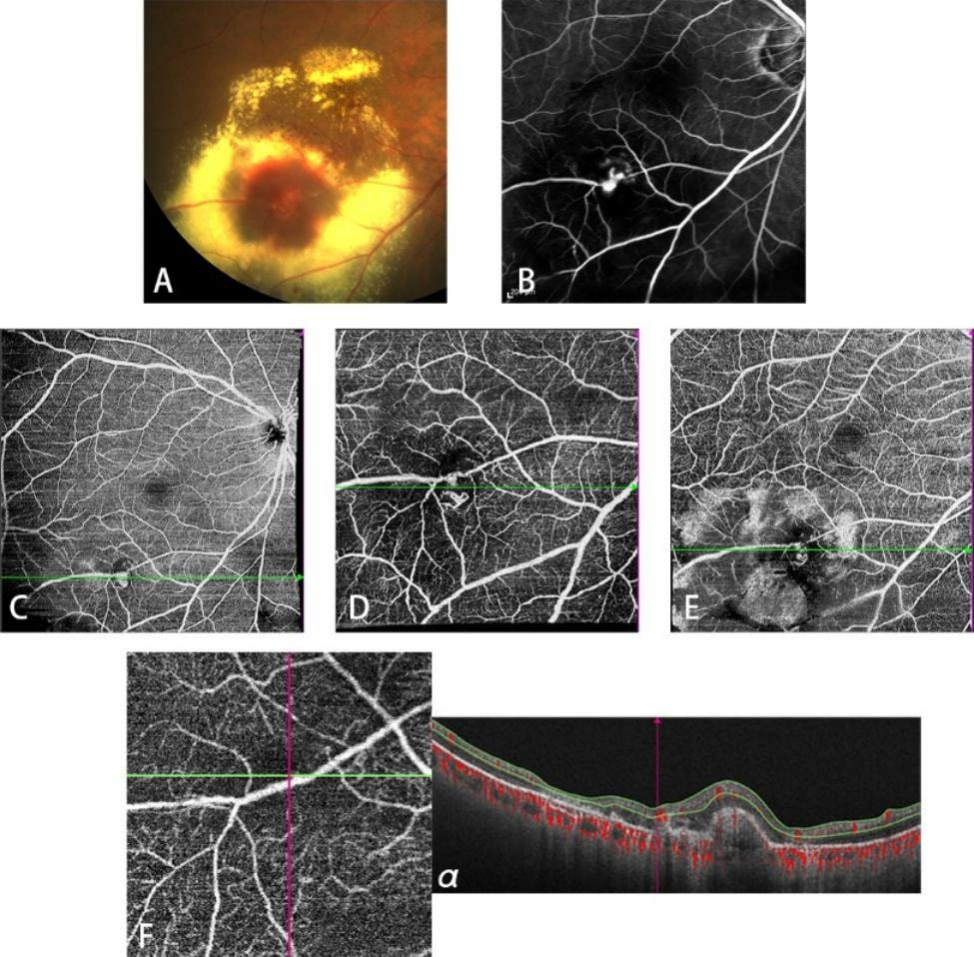
Multimodal Imaging in Patient 9 **A**: Fundus photography before treatment. **B**: FFA before treatment. **C**: SS-OCTA taken 31 days after the 1st anti-VEGF injection. **D**: SS-OCTA taken 36 days after the 1st photocoagulation. **E**: SS-OCTA taken 24 days after the 2nd photocoagulation. **F**: SS-OCTA taken two years after the onset. **α**: B-scan image captured at the green line on F

An 81-year-old woman first received the anti-VEGF injection one month after the onset of symptoms and a photocoagulation the following month. In the fourth month, she underwent another photocoagulation followed by anti-VEGF therapy three days later. Five months after the onset of symptoms, the patient was treated with another injection of anti-VEGF. The last three photocoagulations were given in the sixth, eighth, and ninth months respectively. Initially, the eyeground detected by fundus photography revealed extensive yellowish white exudation at the macula and inferotemporal retina and superficial retinal hemorrhage (Fig. 4A). The characteristics seen were that the RAM was leaking and that the visualized lesion presented as local dilation, and these characteristics could be seen on FFA (Fig. 4B). Compared with the SS-OCTA image taken 31 days after the first anti-VEGF treatment (Fig. 4C), the other two images that were taken 36 days and 24 days after the first and the second photocoagulation, respectively (Fig. 4D, E), did not demonstrate local bulges but demonstrated tangled linear shapes, which are not consistent with the actual morphological changes of the macroaneurysm but correspond to the areas that RBCs were. Anti-VEGF treatment and photocoagulation can both alter the hemodynamics in hemangiomas and cause decreased blood flow and RAM atrophy, while the influence of photocoagulation on hemodynamics is much greater than that of anti-VEGF. Two years after the onset, SS-OCTA showed the disappearance of tangled linear shapes (Fig. 4F) and atrophic RAM (Fig. 4Fα). One month after treatment, the patient’s visual acuity improved from FC/30 cm to 20/200.

## Discussion

RAMs are commonly found at the weaker parts of the arterial walls, such as arteriovenous junctions without adventitial layers and the arteries that are subjected to lipid plaque deposition that destroys the vessel structures. Pathological structural manifestations are prone to emerge due to the inability to maintain their original structures and the decreasing tolerance to pressure. Simultaneously, increased or fluctuating blood flow exerts more uneven pressure on the lesions, so RAMs present as fusiform and saccular. When blood pressure fluctuates greatly or increases suddenly, a hemangioma is likely to rupture. The energy trapped in the lumen pushes blood toward all layers of the retina, and this accounts for the diversity in the clinical manifestations of RAMs. Some patients continue to produce exudate without bleeding due to an increased vascular endothelial cell space. Such exudation relieves the pressure inside the macroaneurysm and thus reduces the risk of rupture and hemorrhage [13]. However, active exudation also threatens the visual function of patients and requires active treatment. In some patients with macroaneurysm rupture, hemostasis by compression occurs due to the blood flowing out of the ruptured vessel, and the hemostasis mechanism, including vasoconstriction and the formation of a clot, is initiated. However, whether the clot can seal up the vessel rupture and fill the lumen to keep the lesion in a static state needs to be examined with angiography and SS-OCTA, which are in favor of instituting therapeutic schedules.

The SS-OCTA detection method is a noninvasive method to detect the movement and trajectory of red blood cells. The molecular weight of sodium fluorescein is much smaller than that of red blood cells and can fill all parts of a RAM, even if thrombi are in the lumen, so the morphology on FFA is closer to the real anatomical structure of RAM. However, if there is turbulence or thrombus in the lesion, red blood cells will advance in a special path or bypass the turbulence or thrombus, thus SS-OCTA records images that can reflect the pathophysiological function of RAM due to the malformation of blood vessels that pipe the blood flow on SS-OCTA. Due to the different appearances and sizes of RAMs and the intervention effect, their flow modes are different, and their trajectories are also different. Some differ markedly from the RAM anatomy in that they are entangled linear shapes. Likewise, it was mentioned in a previous study that polyps are also a cavity, despite them being traditionally considered cystic structures, but they show a linear structure on SS-OCTA [12]. Furthermore, OCTA can also show the structure of RAMs in different layers of the retina to optimize the three dimensional localization of the RAMs [14].

In our study, the morphologies of macroaneurysms could be roughly observed on the SS-OCTA enface, some were locally swollen, some were dilatations on one side or both sides of malformed vessels, some were Z-shaped, and some were not visualized. The fully visualized peaks, similar to the FFA results, show high reflection. The other with incomplete blood flow signals in at least part of the RAMs suggests that there is something, perhaps an embolus, blocking the path of RBCs in the RAM. The disappearance of visible dilatation under different interventions suggests RAM atrophy. This shows that different shapes of RAMs displayed on SS-OCTA are not the simple reflections of the anatomical structures, and all kinds of blood flow trajectories contribute to the diversity of imaging on SS-OCTA. Hu et al. divided treatment-naïve RAMs into four types (i.e., distended, meshed, malformed, and occult types) according to the vascular morphology displayed by OCTA. Of these, the meshed type, which results in neovascularization due to ischemia and hypoxia, is the most frequently observed [15]. Based on the morphology of RAMs we observed, all untreated RAMs were distended types, with 14 presenting as malformed types and 8 as occult types in the later stage, indicating a transition from active to stable lesions. Disease progression and therapeutic interventions can lead to different types of conversions. However, there barely was neovascularization in RAMs we studied. The reported meshed type [15] may be a manifestation of the recanalization of blood flow in the aneurysm, or the establishment of collateral circulation, which requires further clinical observation. Combined with B-scans, SS-OCTA can provide more information about whether the RAM is active or not. A dense and uniform hyperreflection can be seen on the B-scan by scanning beyond the display range on the SS-OCTA enface, but there is no blood flow signal at the corresponding position of the SS-OCTA or B-scan, indicating that what we see on SS-OCTA is only a trace of blood cell flow, which is not equivalent to the real structure of blood vessels. While scanning through the display range on the SS-OCTA enface, where the blood flows, hyporeflective lumen and hyperreflective blood with masking effects inside the lesion can be recorded by B-scan. When the macroaneurysm is large enough, only partial blood flow may be detected on SS-OCTA, and the RAM appearance is not what it truly looks like. These features are related to the velocity and state of blood flow. When there is a thrombus blocked inside, there is no blood flow or small curved blood flow, and some macroaneurysms do not develop thereafter. Not all cysts on SS-OCTA have blood flow. SS-OCTA only represents the detectable movement of blood flow in a certain state, not the realistic appearance shown in fluorescein imaging. It also depends on the machine algorithm and the signal detection range.

By comparing the changes of RAMs on SS-OCTA, relatively rapid changes in the responsiveness after photocoagulations, such as attenuation of blood flow signal, capsular shrinkage, and even subsequent retinal arterial hypoperfusion were observed, whereas these changes were relatively mild after anti-VEGF treatment. OCTA can easily detect these changes after treatment and the revascularization status near the lesion during follow-up. In the future, randomized controlled studies can be conducted to further and more objectively evaluate the prognosis of different treatments.

## Conclusion

SS-OCTA is a noninvasive observation tool, that can be used to observe the filling status of RAMs. This may provide an intuitive basis for further treatment. The blood flow signal does not represent the lumen structure, and will change under different interventions, which can reflect the intuitive changes. Although OCTA does not directly reflect the leakage activity of blood vessels or RAMs, it can evaluate the treatment response and the changes during follow-up through observing the morphological changes. When there is a RAM that is gradually shrinking, fine blood flow, and thrombosis, we can quickly determine whether the lesion has become stable or has faded, especially when these findings are combined with the changes in edema on B-scans. A careful reading of the OCTA images is necessary to assess this progression.

## Data Availability

The datasets used and/or analyzed during the current study available from the corresponding author on reasonable request.

## Abbreviations

RAM: Retinal arterial macroaneurysm
SS-OCTA: Swept-source optical coherence tomographic angiography
FFA: Fundus fluorescein angiography
BCVA: Best-corrected visual acuity
OCT: Optical coherence tomography
SD-OCT: Spectral-domain optical coherence tomography
VEGF: Vascular endothelial growth factor
